# Correlations between the alpha-Gal antigen, antibody response and calcification of cardiac valve bioprostheses: experimental evidence obtained using an alpha-Gal knockout mouse animal model

**DOI:** 10.3389/fimmu.2023.1210098

**Published:** 2023-06-21

**Authors:** Filippo Naso, Andrea Colli, Peter Zilla, Antonio Maria Calafiore, Chaim Lotan, Massimo A. Padalino, Giulio Sturaro, Alessandro Gandaglia, Michele Spina

**Affiliations:** ^1^ Biocompatibility Innovation Srl, Este, Padua, Italy; ^2^ Cardiac Surgery Unit, Department of Surgical, Medical and Molecular Pathology and Critical Care, University of Pisa, Pisa, Italy; ^3^ Christian Barnard Department of Cardiothoracic Surgery, Groote Schuur Hospital, University of Cape Town, Cape Town, South Africa; ^4^ Department of Cardiovascular Sciences, Gemelli Molise, Campobasso, Italy; ^5^ Hadassah University Hospital - Cardiovascular Division, Ein Kerem, Jerusalem, Israel; ^6^ Pediatric and Congenital Cardiac Surgery Unit, Department of Cardiac, Thoracic and Vascular Sciences and Public Health, University of Padua, Padua, Italy; ^7^ Department of Biomedical Sciences, University of Padua, Padua, Italy

**Keywords:** αGal antigen, knockout mouse model, bioprosthetic heart valves, polyphenols, calcification

## Abstract

**Introduction:**

Preformed antibodies against αGal in the human and the presence of αGal antigens on the tissue constituting the commercial bioprosthetic heart valves (BHVs, mainly bovine or porcine pericardium), lead to opsonization of the implanted BHV, leading to deterioration and calcification. Murine subcutaneous implantation of BHVs leaflets has been widely used for testing the efficacy of anti-calcification treatments. Unfortunately, commercial BHVs leaflets implanted into a murine model will not be able to elicit an αGal immune response because such antigen is expressed in the recipient and therefore immunologically tolerated.

**Methods:**

This study evaluates the calcium deposition on commercial BHV using a new humanized murine αGal knockout (KO) animal model. Furtherly, the anti-calcification efficacy of a polyphenol-based treatment was deeply investigated. By using CRISPR/Cas9 approach an αGal KO mouse was created and adopted for the evaluation of the calcific propensity of original and polyphenols treated BHV by subcutaneous implantation. The calcium quantification was carried out by plasma analysis; the immune response evaluation was performed by histology and immunological assays. Anti-αGal antibodies level in KO mice increases at least double after 2 months of implantation of original commercial BHV compared to WT mice, conversely, the polyphenols-based treatment seems to effectively mask the antigen to the KO mice’s immune system.

**Results:**

Commercial leaflets explanted after 1 month from KO mice showed a four-time increased calcium deposition than what was observed on that explanted from WT. Polyphenol treatment prevents calcium deposition by over 99% in both KO and WT animals. The implantation of commercial BHV leaflets significantly stimulates the KO mouse immune system resulting in massive production of anti-Gal antibodies and the exacerbation of the αGal-related calcific effect if compared with the WT mouse.

**Discussion:**

The polyphenol-based treatment applied in this investigation showed an unexpected ability to inhibit the recognition of BHV xenoantigens by circulating antibodies almost completely preventing calcific depositions compared to the untreated counterpart.

## 1 Introduction

Aortic valve disease is one of the most common valvular pathologies (1) with a significantly high mortality rate in symptomatic patients (the actual 5-year mortality with mild aortic stenosis was 40.9% increasing to 52.2% for severe aortic stenosis) (2). The most common cause of aortic valve disease in elderly patients (60-80 years old) is calcific degeneration (3). The treatment of choice is surgical (SAVR, surgical aortic valve replacement) or transcatheter replacement (TAVR, transcatheter aortic valve replacement) according to guidelines (4). Bioprosthetic heart valves (BHV, also known as “tissue valves”) are the most used type of device in more than 80% of all cases worldwide (3). BHVs are made of bovine or porcine pericardium or leaflets valves and are conventionally cross-linked with glutaraldehyde (GA) to ensure tissue stability, reduce antigenicity, and maintain tissue sterility. They are mainly used in patients older than 60 years of age where the durability of the valve exceeds the life expectancy (5). The functioning of BHVs is limited by shorter durability in younger patients in whom the process of calcification is accelerated (6) representing one of the limiting factors for their clinical application.

Traditionally, BHVs calcification has been attributed to extrinsic factors such as the chemical instability of GA, mechanical failure, and intrinsic ones like collagen degradation and calcium precipitation by residual lipids (7, 8). In recent years, the immune-mediate intrinsic pathway gained importance not least because of a better understanding of the αGal xenoantigen trigger (9–11).

Murine subcutaneous implantation of BHVs leaflets has been widely used as an initial step for testing the efficacy of anti-calcification treatments (12–15). However, unlike the situation in humans, bovine pericardial leaflets tissues implanted into a murine model will not be able to elicit an anti-Gal immune response because both donor and recipient species constitutively express αGal epitopes. Some studies have tried to demonstrate the link between the presence of the αGal antigen and the propensity to tissue calcification by comparing pericardial tissues samples obtained from wild-type (WT) and αGal knockout (KO) pigs after the explant from the mice subcutaneous area (16, 17). The αGal KO pig is a genetically manipulated αGal-deficient animal in which the gene responsible for the synthesis of the enzyme α1,3-galactosyltransferase (GGTA1, which catalyzes the αGal saccharide and proteins/lipids bond formation) has been silenced. This genetic modification generates a sort of “humanized” animal that is no longer able to synthesize the αGal similarly to humans thus acquiring in turn the ability to produce anti-Gal antibodies. However, this previous approach (16, 17) is inherently limited as the implantation of biomaterials in WT animals (constitutively expressing the αGal), is precluding any immunological reaction towards the αGal antigen itself (18). Considering what has been reported so far, it seems rational to use genetically manipulated αGal-deficient animals, such as GGTA1-KO mice, as recipient animal models. This could mimic the human immunologic environment, and, to our knowledge, it has not been used to test the efficacy of the treatments of commercial BHVs to prevent the αGal-immune mediated calcification seen in clinical practice (10).

The main objective of this study was to evaluate the amount of calcium deposition in isolated leaflets from the commercial Trifecta-GT BHV model (Abbot/St.Jude, Santa Clara, CA, USA) implanted for 2 months in GGTA1 KO mice and compared with a parallel investigation carried out for up to 4 months in WT (19). Alike and at variance to this parallel report the study was further extended to evaluate the anti-calcification efficacy of a polyphenol-based treatment with specific attention to its ability to mask resident antigens to circulating anti-αGal antibodies. Moreover, we have investigated the level of possible residual αGal-epitope in several tissue districts of the KO-mice considering that a residual amount of αGal epitope reactivity has been recognized in biallelic GGTA1-knockout pig cells and implicated as a possible contributor to chronic rejection of GGTA1^-/-^ organs (20, 21).

## 2 Materials and methods

All animal experiments and surgical procedures were performed in compliance with the Guide for the Care and Use of Laboratory Animals as published by the US National Institutes of Health (NIH Publication 85-23, revised 1996). The use of a mouse animal model for experimental purposes was authorized by the Italian Ministry of Health: project registration number 17E9C.154; authorization number 542/2020-PR. The GGTA1 KO mouse animal model is owned by Biocompatibility Innovation Srl. The cloning was performed in collaboration with Polygene Transgenetic (Rümlang, Switzerland) and the animals are currently housed at Charles River Laboratories Italia (Lecco, Italy).

### 2.1 Cloning of C57Bl/6 αGal knockout mice

All the details are reported in the **Supplementary Material** section.

### 2.2 αGal quantification in WT e KO mouse tissues

Fresh tissue samples from the different tissues (**Table 1**) of WT and KO mice were gently blotted on Whatman filter paper, and their weight was recorded (weight range of about 100mg wet weight). Subsequently, they were incubated with the primary anti-αGal antibody M86 [1:50] (mouse; LSBio, Seattle, WA) for 2hs at 37°C with gentle stirring and finally centrifuged at 14.750g for 30min at 4°C.

**Table 1:** Comparison of the αGal quantification in different tissue districts of wild-type (WT) and GGTA1 KO (KO) mice animal model with the relative percentage of αGal silencing (n=7 for each tissue district).

n° of α-Gal epitope/10mg of tissue			
Tissue/Organ	WT	KO	% of αGal silencing
Eye	7.68*10^11^ ± 0.08	0	100%
Thymus	5.26*10^11^ ± 0.05	0	100%
Tail	4.13*10^11^ ± 0.05	0	100%
Spleen	1.70*10^11^ ± 0.02	0	100%
Myocardium	1.52*10^11^ ± 0.02	2.08*10^10^ ± 0.01	86.3%
Kidney	1.53*10^11^ ± 0.03	2.76*10^10^ ± 0.02	82%
Lung	2.93*10^11^ ± 0.03	5.86*10^10^ ± 0.02	80%
Liver	2.05*10^11^ ± 0.06	4.1*10^10^ ± 0.03	80%
Skin	1.72*10^11^ ± 0.01	9.55*10^10^ ± 0.05	44.4%
Brain	3.35*10^10^ ± 0.02	1.87*10^10^ ± 0.02	44.2%

The number of αGal epitopes was quantified through a patented ELISA test (22). Briefly, a Polysorp 96-well plate (Nunc, Rochester, NY, USA) was coated with 100μl of alpha-Gal/HSA (human serum albumin; Dextra Laboratories, Berkshire, UK), 5μg/ml, for 2hs at 37°C. After washing three times with PBS, the blocking procedure was performed using 300μl per well of 2% HSA (Sigma, St. Louis, MO, USA) in PBS for 2hs, at room temperature in darkness. Wells were then washed three times as mentioned above. A set of wells was loaded with 100μl of supernatant derived from tissue samples of wild-type (WT) and KO mice and incubated overnight at 4°C in darkness. After washing, the secondary HRP-conjugate antibody [1:500] (Dako Cytomation, Glostrup, Denmark) was loaded. Finally, 100μl of horseradish peroxidase substrate buffer was added to each well for 5min at room temperature in darkness. The plate absorbance was measured by a plate reader at 450nm (Multiscan Sky, Thermo Scientific). The number of epitopes was calculated by comparison with a calibration line obtained using rabbit red blood cells (23).

### 2.3 Polyphenols-based treatment of commercial pericardial leaflets

Briefly, a blend of polyphenols was solubilized in phosphate buffer solution (PBS, 50mM NaH_2_PO_4_, 20mM Na_2_HPO_4_) at room temperature as previously described (24–27). Bovine pericardial leaflets isolated from the commercial Trifecta-GT BHV model (Abbott, Plymouth, MN, USA) were allowed to briefly drain, rinsed with PBS and transferred to the polyphenolic reagent solution, and left to react under moderate constant stirring for two consecutive steps of 30min each, at room temperature in the dark. At the end of incubation, the samples were subjected to two washes in isotonic phosphate buffer for 15min each and stored at 4°C in PBS until the moment of implantation. Samples subjected to the polyphenols-based are labeled as F. As already disclosed previously, the chemical interactions between the polyphenols and the tissue constituting the BHVs are investigated by FT-IR spectra and HR-MAS 13C-NMR, resulting mainly in the covalent type with the formation of a very high number of hydrogen bonds (19).

### 2.4 Mice subcutaneous implantation

Calcium quantification at 4 months in WT animals, performed in a parallel investigation (20), demonstrated a non-statistically significant difference between the amount of calcium quantified after 2 and 4 months of tissue implantation in the subcutaneous back area. Considering therefore the WT mouse as the primary reference, it was reasonably decided to consider an implantation time not exceeding 2 months. Alike and at variance to the parallel investigation (20), GGTA1 KO mice (C57BL/6, 6 weeks old, 30g) instead of WT mice were used.

After anesthetizing and shaving, a subcutaneous pouch was created in the dorsal area for each mouse. Each not-treated (NT, n=16) and polyphenols-treated (F, n=16) Trifecta-GT leaflet was implanted into the pouch of each animal, and the wounds were closed with 6/0 nylon sutures. After 1, and 2 months the mice were sacrificed under a CO_2_ atmosphere and the samples were carefully harvested.

### 2.5 Evaluation of anti-αGal antibodies production in KO mice

Anti-αGal serum IgM and IgG antibodies from the αGal KO mice were determined before and 2 months after implantation of original and polyphenols-treated leaflets from Trifecta-GT, by enzyme-linked immunosorbent assay (ELISA). About 0.5 – 1.0ml of blood per mouse was collected by infraorbital venous plexus sampling (n=10). A Polysorp 96-well plate (Nunc, Rochester, NY, USA) was coated with 100μl of alpha-Gal/HSA (Bovine serum albumin; Dextra Laboratories, Berkshire, UK), 5μg/ml, for 2hs at 37°C. After washing three times with PBS, the blocking procedure was performed using 300μl per well of 2% HSA (Sigma, St. Louis, MO, USA) in PBS for 2hs, at room temperature in darkness. Wells were then washed three times as mentioned above. A set of wells was loaded with 100μl of [1:80] diluted serum and incubated overnight at 4°C in darkness. After washing, the secondary HRP-conjugate anti-mouse IgM and IgG antibody [1:500] (Jackson Immunoresearch, Pennsylvania, USA) were loaded. Finally, 100μl of HRP substrate buffer was added to each well for 5min at room temperature in darkness. The plate absorbance was measured by a plate reader at 450nm (Multiscan Sky, Thermo Scientific).

### 2.6 Calcium quantification in explanted commercial leaflets

Polyphenols-treated (F) and non-treated (NT) leaflets from Trifecta-GT BHVs were carefully explanted from KO mice and washed twice in sterile cold PBS for 10min. Specimens were subsequently subjected to acid hydrolysis in HCl 6N at 110°C for 12hs. Calcium evaluation was performed in hydrolyzed samples by inductively coupled plasma according to the directives of the EPA6010D method (28) and expressed as µg Ca^2+^/10mg of dry defatted weight (ddw). As a control sample, calcium quantification was also carried out in unimplanted off-the-shelves original Trifecta GT™ valve leaflets.

Ddw was determined by comparing lyophilized dry-weight samples before and after delipidation treatment. After the lyophilization step, sample tissues were incubated for 36hs under 10 kPa over P_2_O_5_ at 37°C until a constant dry weight was attained. The defatted procedure was carried out by incubation of tissue specimens in ascending series of alcohols followed by two steps of chloroform/methanol (2:1 and 3:1 v:v), in a descending series of alcohols, and finally in the water.

### 2.7 Von Kossa staining in explanted commercial leaflets

Representative polyphenols-treated (F) and non-treated (NT) tissue samples explanted from WT (at 4 months of follow-up, n=4) and KO (after 2 months of follow-up, n=4) mice were carefully rinsed with cold PBS and subsequently embedded in OCT compound (Tissue Tek; Sakura Finetek, Tokyo, Japan), cryo-cooled in liquid nitrogen, and cut into 8μm cryosections. Sections were stained with Von Kossa. The general appearance of the extracellular matrix (ECM) and calcium deposition were examined.

### 2.8 Statistical analysis

The data were analyzed in Microsoft Excel^®^ and Prism^®^ 7 for Windows (v7.03, GraphPad Software lnc., California) and expressed as mean ± standard deviation (SD). A two-sided unpaired T-test was used to assess significant differences between the treated and untreated groups, at the 0.95 confidence level.

## 3 Results

### 3.1 αGal quantification in WT e KO mouse tissues

As reported in **Table 1**, the GGTA1 gene silencing inhibited αGal antigen synthesis in a non-uniform manner, ranging from 100% to 80%, depending on the tissue district. For both the skin and the brain samples, the inhibition of the antigen expression was limited to 44%. Interestingly, the brain of the WT mouse exhibits the lowest number of αGal antigens while accounting for the same epitope amount determined in the KO mouse after gene silencing.

### 3.2 Evaluation of anti-αGal antibodies production in KO mice

**Figure 1:**
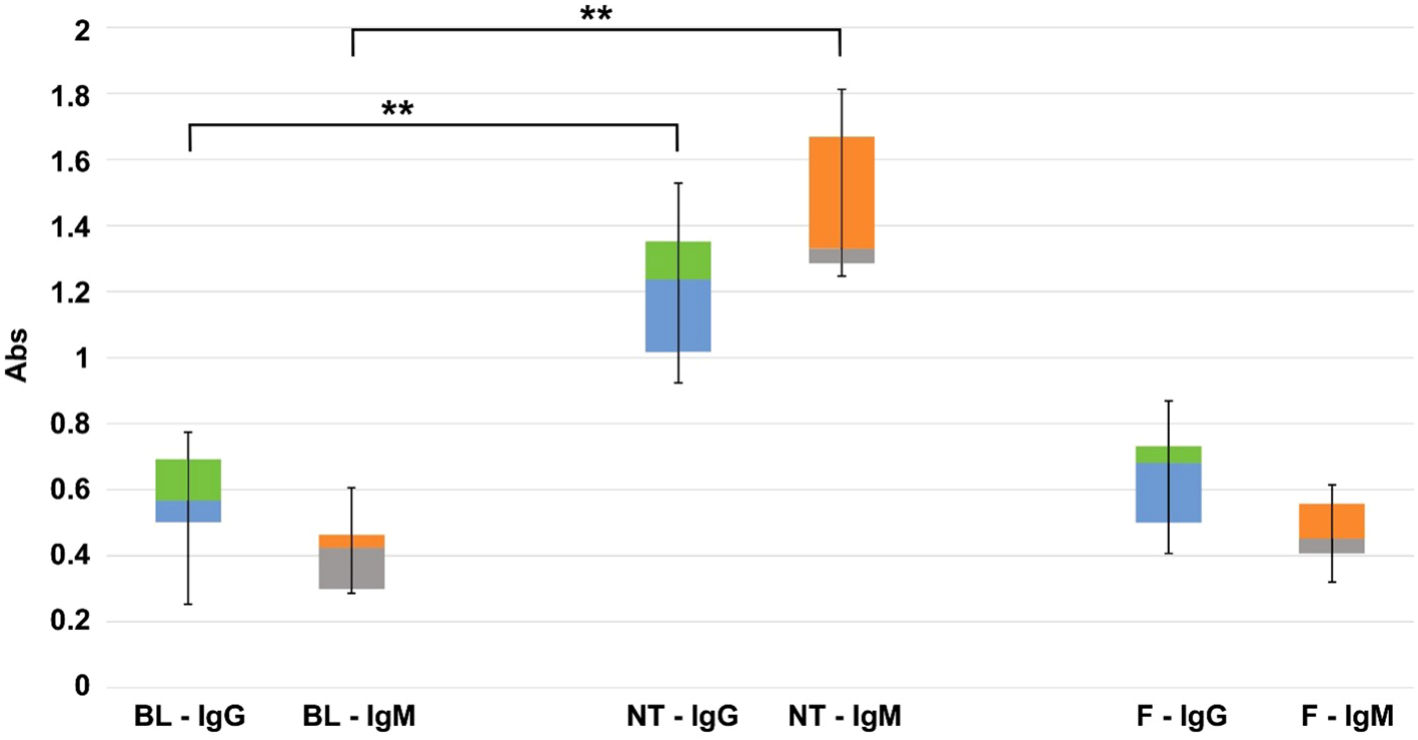
Quantitative evaluation (450 nm OD absorbance units) of IgG and IgM anti-Gal antibody production in KO mice. On the left the basal level (BL), in the center, and on the right the variations found after 2 months of implantation of commercial Trifecta-GT valve leaflets not-treated (NT) and polyphenols-treated (F) (n=10 for each type of sample including BL). The data points represent the means ± SD. ** Indicates a statistically significant difference between the two groups at the 0.95 confidence level.

As a result of food intake during housing, the GGTA1 KO mice develop a bacterial flora expressing the αGal antigen, thus leading to the onset of a baseline level of IgG and IgM anti-αGal antibodies. This baseline level appeared to be further increased following the implantation of the Trifecta-GT leaflets (comprising glutaraldehyde-treated bovine pericardium) previously reported to contain a significant amount of this antigen (11). In this specific case, the 2 months of permanence of the implants in the mouse subcutis, doubled the level of circulating anti-Gal IgG and more than tripled that of IgM (**Figure 1**, BL vs NT). Particularly, the polyphenol-based treatment demonstrated the ability to make the treated tissue “undetectable” to the mouse’s immune system while preventing the increase of anti-Gal antibody levels otherwise observed in the case of NT samples implantation: there were no statistically significant differences between the IgG and IgM baseline levels found after 2 months of implantation of the polyphenols-treated leaflets (**Figure 1**, BL vs F).

### 3.3 Calcium quantification in explanted commercial BHV leaflets

**Figure 2:**
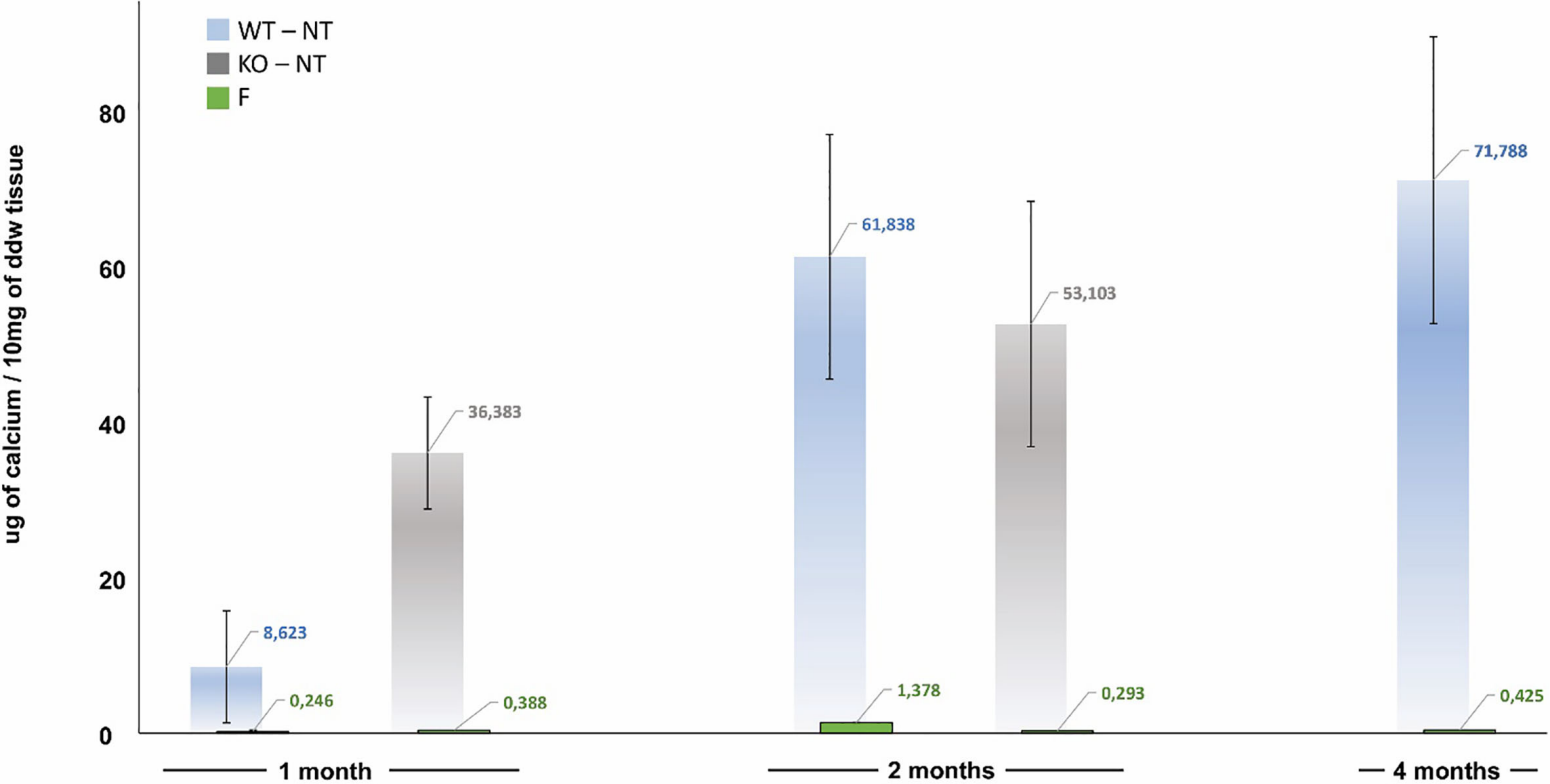
Calcification trend in not-treated (NT) and polyphenols-treated (F, green bar) currently adopted leaflets of Trifecta-GT implanted in the subcutis back area of wild-type mice (WT, light blue bar at 1, 2, and 4 months of follow-up) and knockout for αGal antigen (KO, grey bar at 1 and 2 months of follow-up). As a control sample, calcium quantification was also carried out in un-implanted off-the-shelves original Trifecta GT™ valve leaflets resulted to be 1.19 ± 0.05 µg/10mg of ddw.

In Trifecta-GT leaflets explanted from the KO mice, a relevant calcium deposition (**Figure 2**, grey bar) was evident even after one month and accounted for more than four times the amount found in the WT mouse at the same time (1 month KO *vs* 1 month WT p=0.015). The intensity of mineralization increased after two months, it was not significantly different from that determined in the WT mouse at the same time and comparable to that determined in the 4-month WT model. Again, similarly to what was already evidenced in the WT mouse, the polyphenols-based treatment exhibited a strikingly evident anti-calcification effect even in the KO-F samples. The surprisingly efficient treatment with polyphenols appears to prevent by over 99% the calcium deposition in both WT and KO animal models.

### 3.4 Von Kossa staining in explanted commercial leaflets

**Figure 3:**
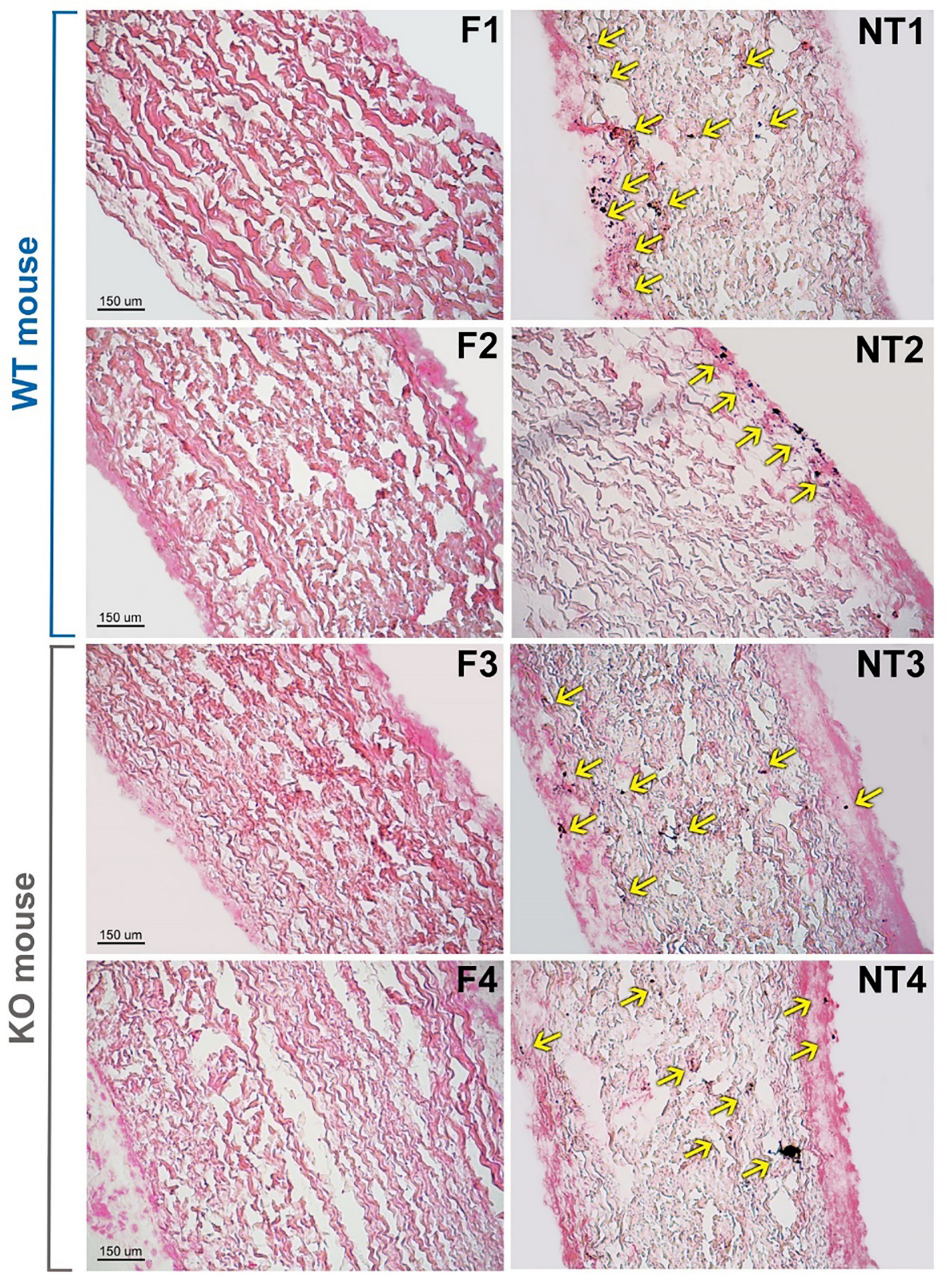
Histological evaluation of calcium deposition in representative not-treated (NT) and polyphenols-treated (F) leaflets from Trifecta-GT valve implanted in the subcutis back area of wild-type (WT, 4 months of follow-up) and αGal knockout (KO, 2 months of follow-up) mice. Spots of calcified deposition are highlighted by yellow arrows. Von Kossa staining, magnification 10X.

The histological evaluation of calcium deposition, in some representative explanted leaflets (**Figure 3**), was found to be in line with the results of plasma analysis (**Figure 2**). In general, the non-treated leaflets (NT) exhibited unevenly diffused micro-calcifications. Their counterparts, treated with polyphenols (F), did not show calcified spots even when calcium content accounted for about 0.3 μg/10mg of ddw. It is known that the sensitivity of the quantitative Inductive Coupled Plasma technique is considerably higher than the histological evaluation. Particularly, the calcium content of the F leaflets resulted below the detection limit of the Von Kossa staining, besides the fact that, unexpectedly, it was even significantly lower than that determined in the untreated control group samples before implantation.

## 4 Discussion

GA is often used as the preferred fixative and sterilizing agent for many commercial bioprosthetic products, unfortunately, the GA chemical instability is strictly involved in the exposure of potential calcium-binding sites (residual aldehydes, acids, Schiff bases, etc). As a result of the interaction between tissue amino acid residues and GA, negatively charged carboxylic acid groups can be created which can electrostatically interact with positively Ca^2+^ charged ions, becoming a tremendous attraction site for calcium. To make matters worse, even the free-to-react aldehyde groups can be easily oxidized into carboxylic residues via air, *in-vivo* blood, and macrophage oxidation. To decrease the influence on the calcification process, several changes in the GA fixation protocols have been proposed by the BHVs manufacturers, including the addition of novel steps aimed at the chemical stabilization of the reactive aldehyde and carboxylic groups. GA detoxification by urazole, diamine spacer extension, treatment by 2-amino oleic acid, or incubation in ethanol is just some of the processes developed in the challenge of stabilizing GLU, with the hope of delaying the calcific tissue dystrophy (29). Although the calcified degeneration of the BHVs is the long-term event generally responsible for the definitive failure of such biomedical devices, it must not be forgotten that there are a series of degenerative processes that begin to affect the prosthesis just a few hours after implantation. It is now well established as such degenerative active mechanisms are triggered by early host’s immune-response toward the implant (19). Recently, the results of the Translink international collaborative study group have been released (10). Translink is a prospective European Union-funded collaborative project, which assessed the role of the xenoantigens in BHVs deterioration. In particular, Translink is focused on the involvement of anti-glycan antibodies in inducing calcification of BHV tissues, confirming that BHV xenogeneic antigens contribution to the immunogenicity of animal-derived implants, is eliciting antibodies that are likely involved and support valve calcification. The results obtained from this study provide evidence that the lack of the αGal epitope in the GGTA1 KO mice was associated with an early (one-month) response leading to quadrupling the calcium deposition rate determined in WT animals at the same time (**Figure 2**). This initial response is recalling the severe calcific deposits associated with the early failure of porcine heart valve transplanted in pediatric patients (30) and successively suggested to be related to the presence of residual αGal epitopes (31).

Such residual presence of αGal xenoantigen increases the human anti-galactose titers, starting from day 10 following BHV implantation (32) while reaching a peak at around 3 months (33) for IgM (+45.1 ± 10.5%) and IgG (+21.7 ± 4.65%) isotype. The increase in the production of anti-Gal antibodies does not decrease even 5 years after implantation (34), confirming that a basic immune-stimulation is always active, probably due to the chemical instability of the GA which leads to the exposure of previously masked antigens over time. This sugar moiety is expressed in most mammalian tissues, except humans and higher primates. In humans, the continuous antigenic stimulation by gastrointestinal flora (expressing the αGal epitope) results in the production of anti-αGal antibodies accounting for 1 to 3% of the circulating immunoglobulins. Different research groups (10, 35) have demonstrated that these preformed antibodies could cause opsonization of the valve tissue with consequent initiation of specific Fc-receptor-mediated macrophage recruitment with antigen processing and presentation, resulting in extracellular matrix (ECM) calcification and deterioration.

In agreement with what was reported in humans and available in the literature (10, 32–34), (tissues in which masked xenoantigen carbohydrates, do not trigger antibody-mediated calcification), the anti-αGal antibodies in the GGTA1 KO blood analysis resulted in a remarkable IgG and IgM increase only in mice that received a bioprosthetic leaflet not treated with polyphenols (**Figure 1**). Accordingly, the use of polyphenols results in a powerful approach able to prevent calcium deposition as assessed in two different mouse animal models. Of interest that polyphenols have also been reported to inhibit calcium deposition in BHVs when tested in an *in vitro* system (24).

Small animal models such as rats or mice are widely used for *in vivo* biomaterial assessment for their low cost, ready availability, ease of handling, and well-defined immune parameters. These models are generally used for the assessment of chronic changes to BHV leaflets implanted in an ectopic (non-cardiac) location. In particular, the subdermal model provides permanent contact of the implant to host tissue and sufficient blood supply (serum exposure), which eases cellular infiltration and allows a rapid screening efficacy for anti-calcification treatments.

Particularly, the use of the GGTA1 KO animal model enables a differential evaluation of the immune-mediated effects of the αGal concerning that of the whole of other intrinsic and extrinsic factors leading to the calcification of BHVs (as also revealed by the WT mouse model). In fact, besides the early calcium deposition, the increase of anti-αGal IgG and IgM, specifically due to the presence of the αGal epitope in the implanted tissues, is opening the way to the separate detection of residual αGal antigen in any kind of implantable biomaterials. In addition, the results of this investigation are further confirming the presence of immunologically active αGal antigens in BHVs currently adopted in clinical practice as previously determined, by a different analytical approach (11).

**Figure 4:**
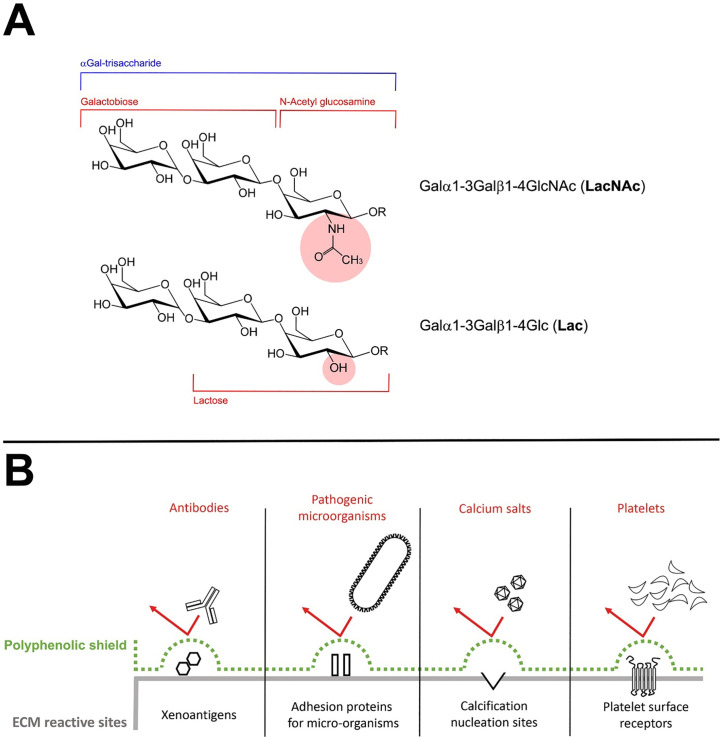
**(A)** Structure of the different αGal xenoantigenic trisaccharides. In particular, the portion of the structure corresponding to the Galα1-3Gal (galactobiose), the N-Acetyl glucosamine (NAc) and the Lactose (Lac) has been clearly identified. The presence in the molecular structure of the NAc or the Lactose group is responsible to determine the immunogenicity of the global trisaccharide. **(B)** The polyphenolic tri-dimensional network sterically covers and shields the recognition of specific extracellular (ECM) reactive sites physiologically involved in triggering the degenerative phenomena affecting the BHVs after the implant.

Formation of the αGal epitope mostly occurs by the transfer of galactose in a α(1,3)-glycosidic linkage to an N-acetyllactosamine (LacNAc) acceptor molecule group present on protein and lipid (**Figure 4A**). This reaction is encoded by the GGTA1 gene (36). However, a residual amount of αGAL epitope reactivity has been recognized in biallelic GGTA1-knockout pig cells and implicated as a possible contributor to chronic rejection of GGTA1^-/-^ organs as found in non-primate models of xenotransplantation (20, 21). The existence of another Gal-transferase in GGTA1 KO mice was already reported by Milland et al. (37) identifying isoglobotrihexosylceramide synthase (iGb3s) in GGTA1 KO mice and the presence of a small amount of iGb3s in tissues using monoclonal antibodies. IGb3S is known to generate the Galα1,3Gal disaccharides epitopes on glycosphingolipids (Lac) by the addition of galactose to lactosylceramide (**Figure 4A**).

The comparison of the αGal epitopes number as quantified in the different tissues of WT and GGTA1 KO mice confirmed what has already been reported in the literature namely the presence of variable antigenic residues despite the silencing of the GGTA1 enzyme (**Table 1**). To our knowledge, this is the first report, to provide the distribution of the residual percentage of αGal likely related to the enzyme iGb3s compared to the uppermost activity of GGTA1.

The αGal epitope is related to the iGb3s enzyme exclusively bound to lipid components, in particular, to ceramides. In the mammalian nervous system, nerve conduction is facilitated by myelin, a lipid-rich membrane that wraps around the axon. The myelin sheath is a specialized structure with distinct lipid and protein constituents. Galactosylceramide (GalCer) and sulfatide make up approximately 30% of total myelin lipids (38). In particular, the levels of GalCers are especially high in the brain and have been reported to be higher than glucosylceramides (GluCers) in the WT mouse brain (39). Previous studies revealed that ceramides mediate signal transduction and cell adhesion and are crucial for the formation of nervous tissues (40), this could explain why even in the KO mouse a significant amount of iGb3s-αGal epitopes are available, due to their unavoidable presence for correct brain and neuronal function. The outermost layer of the mammalian epidermis is the stratum corneum, which is made of flattened, enucleated keratinocytes and a unique extracellular lipid matrix produced by differentiating keratinocytes. The stratum corneum provides the permeability barrier against water and various environmental agents, such as chemicals and microorganisms. About half of the lipids in the stratum corneum are mixtures of ceramides (41). Significant levels of ceramides were also found in the kidneys, liver, lungs, and myocardium (42). However, the residual percentages of αGal quantified in this study, agree with what was reported by Shao A and colleagues who describe a reduction in the expression of the antigen between 5.19% and 21.74% in GGTA1-KO mice (18), except for the skin and brain areas where, due to a higher concentration of ceramides, the reduction is much smaller.

Noteworthy, GGTA1 KO animals do not seem to express iGb3s in sufficient amounts to mediate cell destruction: the work of Murray and colleagues reports as a minimum threshold of αGal expression is required to induce antibody-mediated skin graft rejection in a mouse GGTA1 KO model (43). Accordingly, a previous study demonstrated that silencing the porcine iGb3S gene did not affect measures of anticipated pig-to-human and pig-to-primate acute rejection, suggesting iGb3S is not a contributor to antibody-mediated rejection in pig-to-primate or pig-to-human xenotransplantation (44). In fact, the αGal contribution due to the presence of iGb3S is not appreciable by heat-inactivated human and baboon sera antibodies when incubated with GGTA1 KO or GGTA1/iGb3S double KO pig tissue. This is the reason, even if residues of αGal antigen are still present, the αGal KO mouse was revealed as an adequate animal model for evaluating the calcification propensity of currently adopted BHV tissues. The implantation of Trifecta-GT valve leaflets has significantly stimulated the mouse immune system and resulted in massive production of anti-Gal antibodies (**Figure 1**) both IgM and IgG type. This mouse immune-mediated reaction is apparently quite similar to that occurring in humans, as extensively reported in the literature (32, 33). All that raises important considerations in evaluating the efficacy of anti-calcific treatments, making it clear that the choice of small animal models must necessarily prefer the KO model in order not to incur a possibly dramatic underestimation of the potential triggered by immune-mediated reactions towards xenoantigens.

What is more, polyphenols are known for their strong anti-inflammatory potential (45) and for being effective in masking xenogenic antigens as previously reported (24). In particular, in this study, the polyphenols-based treatment confirmed an unexpected ability to inhibit the recognition of BHV xenoantigens by circulating antibodies even in a GGTA1 KO mouse while almost completely preventing calcific depositions compared to the untreated counterpart. The effectiveness of such treatment is furtherly supported by the already disclosed ability of polyphenols to reach an unprecedented chemical stabilization of the GA, remarkably enhancing the inactivation of the free-to-react carboxylic and aldehyde groups to 76% and 55%, respectively (19). Concluding the investigation on the chemical interaction between polyphenols and ECM (19) has highlighted how polyphenols can interact forming a three-dimensional network (both internally and around the tissue) sterically covering and shielding the recognition of specific extracellular matrix reactive sites (xenoantigens (24), adhesive sites for pathogenic micro-organisms (27), calcium nucleation sites and platelet surface receptors (19)) physiologically involved in triggering the degenerative phenomena affecting the BHVs after the implant (**Figure 4B**). Noteworthy, this network is not an insulator but allows the exchange of water, ions, and various pro-active substances between the inside and outside of the matrix. The protective effect of polyphenols can therefore be reasonably considered as a synergistic action of various factors which, acting on different levels, converge in the results in inhibiting the degenerative mechanisms, including calcification.

## Data availability statement

The raw data supporting the conclusions of this article will be made available by the authors, without undue reservation.

## Ethics statement

The animal study was reviewed and approved by Italian Ministry of Health: project registration number 17E9C.154; authorization number 542/2020-PR.

## Author contributions

FN contributed to the conception and design of the study, writing the original draft; AC, PZ, AMC, and CL validation; MP, GS, and MS writing, review and editing; AG project administration and funding acquisition. All authors contributed to the article and approved the submitted version. 
