# Supplementary material for: Correlations between the alpha-Gal antigen, antibody response and calcification of cardiac valve bioprostheses: experimental evidence obtained using an alpha-Gal knockout mouse animal model
Source: Front Immunol. 2023 Jun 21;14:1210098. doi: 10.3389/fimmu.2023.1210098 (PMC10327888; doi:10.3389/fimmu.2023.1210098)
Supplement: Supplementary file 1 [file DataSheet_1.pdf]

Polyphenol-based treatment decreased the calcification of commercial bioprosthetic heart valve in humanized murine αGal knockout animal model

SUPPLEMENTARY MATERIALS

S1. INTRODUCTION

PolyGene I139 is a CRISPR-based targeting project for the constitutive knockout of glycoprotein galactosyltransferase alpha 1, 3 (Ggta1). Ggta1 is a cell surface-anchored enzyme that transfers galactose and is implicated in several processes of cell adhesion and activation. Despite an apparent role in gonadic maturation and fertilization, knockout mouse strains have been generated before, and have healthy breeding behavior. The gene also has a modulatory effect on metastasis and cancer.

Ggta1 in mice has a few expressed transcripts involving different exons, and in some cases, polyadenylating at different sites (see Figure S1). The current goal is to inactivate the gene in a manner that none of these transcripts are made into a functional peptide.

S1.1 Targeting strategy

The structure of the gene offers a very fortunate strategy to eliminate all of the translated messages: exons 4 and 5 of Ggta1-202, resp. 5/6/7/8 of Ggta1-203, or 5/6/7 of Ggta1-204, or 5/6/7 of Ggta1-205 (i.e. all relevant exons downstream of the second, and always non-skipped translated exon: number 3 of Ggta1-202), have nucleotide numbers that are a multiple of three. This means that any frameshift mutation in that exon will lead to a protein with a scrambled code (a frameshift in the ATG exon, Ggta1-202_2, could still be compensated by skipping). We, therefore, target exon Ggta1-202_3. For this, we clone guide sequences into plasmid vectors that express the single-guide /synthetic-guide RNA (sgRNA) as well as the Cas9 protein. These injections are usually highly efficient.

The CRISPR/Cas9 system consists of three major elements: A gRNA responsible for site recognition in the genome, the Cas9 protein which introduces the double-strand break in the DNA, and a so-called tracrRNA which mediates the interaction between the gRNA and Cas9 (for review see Hsu et al., 2014, Vol 157(6), p1262–1278). Together the gRNA and the tracrRNA build up the guide RNA (sgRNA). To design gRNAs for Ggta1, we used the publicly available algorithm from the Institut des Neurosciences (http://crispor.tefor.net). The algorithm identified quite a few possible guide RNA sequences, despite high homology between the actin genes and pseudogenes. The identified guide sequences have a high score, accounting for their specificity; also, a list of eventual off-targets is provided and these can be inspected. Guide RNA sequences are defined as 20 bp of gene-specific sequence followed by a so-called Protospacer Adjacent Motif (“PAM”; this sequence is NGG for Cas9). Two sgRNAs were selected based on this algorithm for the Actb gene (see Figure S2). The guide RNA sequences were blasted against the mouse genome for off-targets. The analysis by the algorithm weighs up different off-targets by the number of mismatches and the position of mismatches and subtracts this number from 100. A score between 100 and 50 is recommended for specific CRISPR/Cas9 action. Both guide sequences have a score in this range.

S2. MATERIALS and METHODS

S2.1 Pronuclear CRISPR Microinjections

Pronuclear microinjections were performed using the above guides cloned into an expression plasmid expressing all elements of the CRISPR/Cas9 complex; guide plasmids for both guides were cloned and used. However, after two injection sessions with unsatisfactory outcomes, we decided to switch strategies performing the model via targeted ES cells, generating several genetically identical founder mice.

S2.2 Targeting in ES Cells

ES cells (60'000 / 120'000 on 6 cm plates) were lipofected (Invitrogen Lipofectamin LTX) using 1 µg of the guide plasmid, and 1 µg of the homology oligo in each reaction. After 24 h, the cells were selected for 48 hours with 0.8 µg /ml puromycin (to select for the transient expression of the guide/Cas9 plasmids) and then picked after 8 days (2 x 48 clones from each guide). Candidate clones with visibly reduced amplicon size upon PCR screening (see below) were expanded to 24-well format and frozen at -80°C, retested by PCR, and sequenced. The yield of clones with visible deletions was low, but we nevertheless identified 6 candidate clones (see Figure S4). PCR on the clones was performed using the primers:

I139.5 5'- GGATGCTGGGAACTGAATCG-3'

I139.6 5'- AGGAGCACGGCATGAAAG-3'

The screening PCR with primers I139.5/6 yields a wild-type amplicon of 436 bp, small enough to identify small deletions, yet not too small to miss deletions of up to 2-300 bp (Figure S4). Sequencing of the candidate clones ultimately yielded:

Clone 3C8 34 bp and LOH (Loss of Heterozygosity)

Clone 3C12 wild type

Clone 3D7 71 bp deletion & wild type

Clone 4E3 110 bp deletion and LOH

Clone 4E7 75 bp deletion / 2 bp deletion

Clone 4E8 32 bp deletion and LOH

LOH resembles homozygosity and is in CRISPR generation often mistaken for it. In the above cases, it is obvious that peculiar variants/mutants are extremely unlikely to happen twice on corresponding alleles, and therefore probably represent a preferential amplification of the mutant and hence seemingly mutant-specific sequence information. It does indeed seem as if the wild-type band or at least a band with a similar size to wild type is indeed present in 3C8, 4E3, and 4E8 (see Figure S4), albeit weaker.

S2.3 Blastocyst injections of ES cell clones

Five candidate clones were injected into C57Bl/6Ng blastocysts on May 30th and 31st, 2019, (see summary in Table 2). The injections were highly effective, yielding several 80-100% chimeric males from each litter born in three of the five clones (see Table S1).

S2.4 Germ line breeding and genotyping

Eleven of the chimeric mice obtained from the blastocyst injections were mated to C57Bl/6Ng mice to assess transmission to the germ line. Although we did obtain offspring from 3C8 mice, we didn't pursue this any further, as these mice did not happen to breed very well and were soon behind the very successful breedings of the other clones, and because of the deletion size that seemed to us more appealing in the others.

Surprisingly, mice from 3D7 were segregated into two sublines, one with the characterized 71 bp deletion, and one with 18 bp. All of the pups had either deletion, pointing to a clean heterozygous constellation. The 18 bp deletion was within exon 4 but 55 bp downstream of the 71 bp deletion start, and hence not visible in the overlap PCR (see Figure S5). On the other hand, 18 bp is a small enough difference not to be safely identified on the agarose gel (see Figure S4). Hence, at some point, we mistook it for the 2 bp deletion in clone 4E8 and bred it anyway, but later eliminated all respective mice. A later inspection clarified that 4E8 had not yielded chimeric offspring. Screening for the deletions was performed using primers I139.5 and I139.6, an example of it is shown in Figure S6. Breeding with 71 bp deleted mice from clone 3D7 and 75 bp deleted mice from clone 4E7 was very productive and yielded a Mendelian distribution in D71 (20/42 mice; all others D18) as well as from D75 (23/40).

Several pairs of D71 and D75 mice were set up for breeding to generate homozygous F2 mice. To date (Feb 25, 2020), 227 F2 mice were generated. Of those, 152 were from the D71 and D75 breedings. The ratios in these mice were: D71: 15/37/17 (wt/het/hom); D75: 23/39/21 (wt/het/hom). These are fairly accurate Mendelian ratios and suggest that there is no effect on development or viability for the mutant heterozygous or homozygous genotype. To reconfirm the genotypes, we sequenced several

homozygous mice using the I139.5/6 PCR and I139.5 as a Sanger sequencing primer, confirming the sequencing data obtained in ES cells.

S3. DISCUSSION

Generating deletions using CRISPR technology in Ggta1 was highly efficient, and the seeming detour via ES was a wise choice with the high rates of qualitative mutants. The large, non-multiple-of-three deletions that can be chosen from are an additional advantage; the standard outcome of CRISPR-based deletions are small deletions causing a frameshift, but there is accumulating evidence that this is a risky strategy: small frameshifts can be compensated by misreading.

The two used genotypes (D71, and D75) represent full functional knockouts. In D71, an out-of-frame deletion within exon 4 causes a frameshift, and all ensuing exons all multiples of 3; hence, the reading frame cannot be taken up again, a message starting from the genuine ATG is completely ablated. In D75, splicing into exon 4 causes a runaway message that will probably somewhere encounter a stop or a weak splice donor, and most likely cause a message that is rapidly cleared out.

TABLE

Injections	Clones	Blast Injected	Blast transferred	Fosters	Pups
I139.8	4E7, 4E8, 4E3	61	58	3	10
I139.9	3C8, 3D7	41	40	2	12
**TOTAL**		**102**	**98**	**5**	**22**

Table S1 - Summary blastocyst injections with I139 clones. Five candidate clones were injected on 2 different occasions. The injections were exceedingly successful and effective, yielding 20 out of 22 highly (80-100%) chimeric mice, with just one of them female, from clones 3C8, 3D7, 4E7.

FIGURES

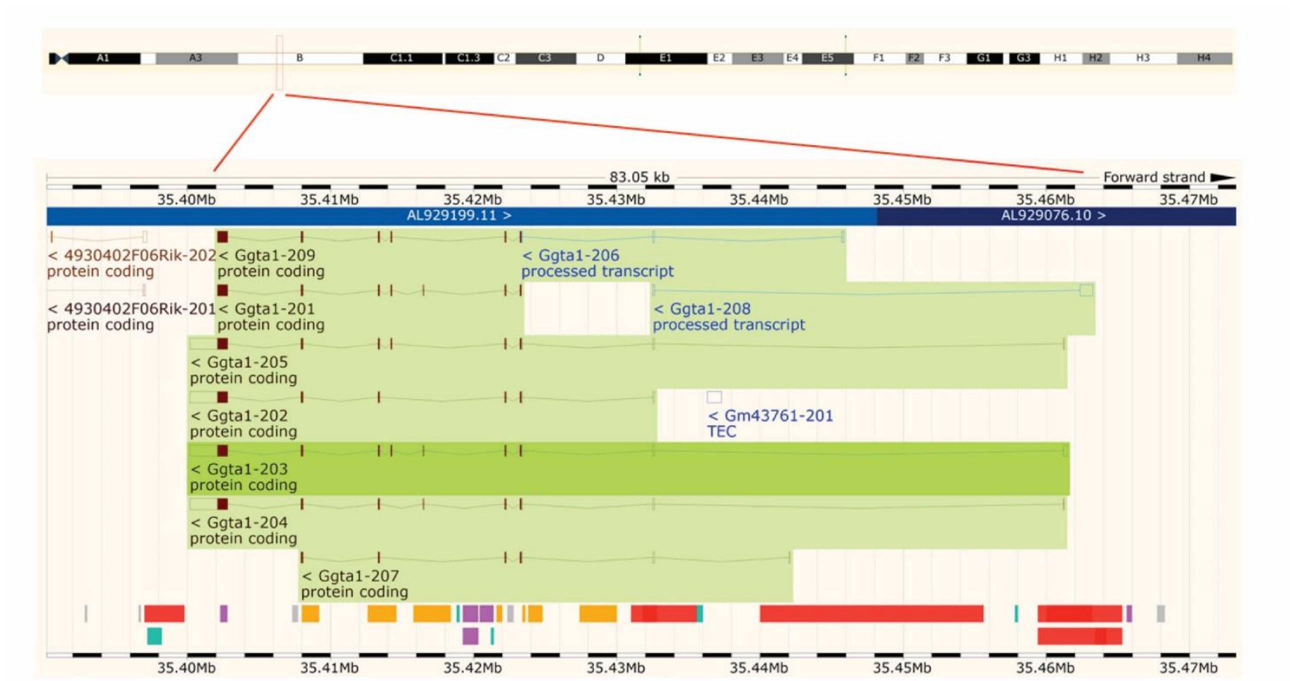

Figure S1 - Location of the Ggta1 gene on mouse chromosome 2. The gene is in counter orientation and is represented by several peptide coding transcripts.

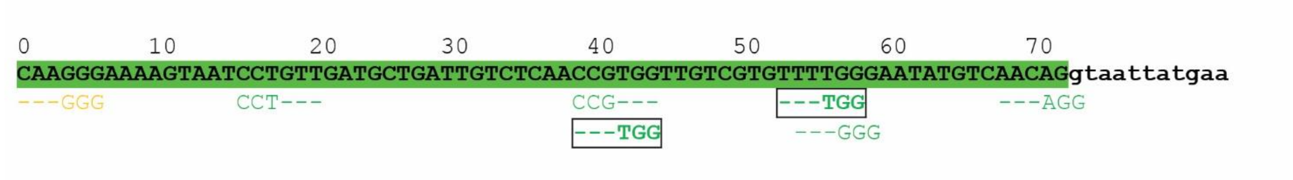

Figure S2 - End region of mouse Ggta1-202 exon 3 and possible CRISPR guide sites. The coding region of exon 3 is shown on green background, possible CRISPR guides are shown below the sequence are the PAM site and the expected cleavage position located -3bp 5' of the PAM site. Colors green, orange, and red indicate high, medium, and low specificity of the PAM's guide sequence in the genome.

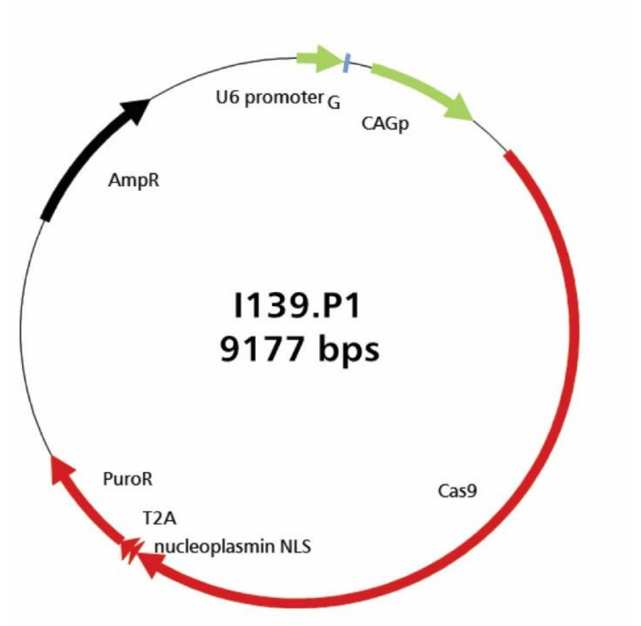

Figure S3 - I139.P1 All elements of the CRISPR/Cas9 complex are expressed from this vector. I139.P1 contains the I139.sgRNA1. Vector I139.P2 (not shown) only differs in this part, the latter contains sgRNA2.

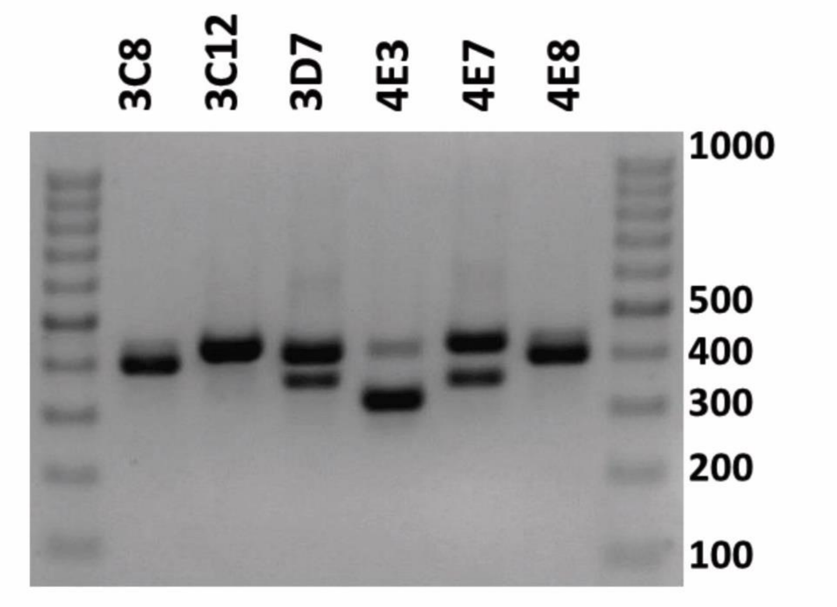

Figure S4 - PCR on Candidate Clones. Expanded clones were re-prepped and amplified with the screening PCR (I139.5/6). A wild-type control is not included but there should be a top band, only clearly visible in 3D7 and 4E7, corresponding to the native allele. Upon sequencing analysis, 3C12 turned out wild-type and therefore shows the 436 bp band.

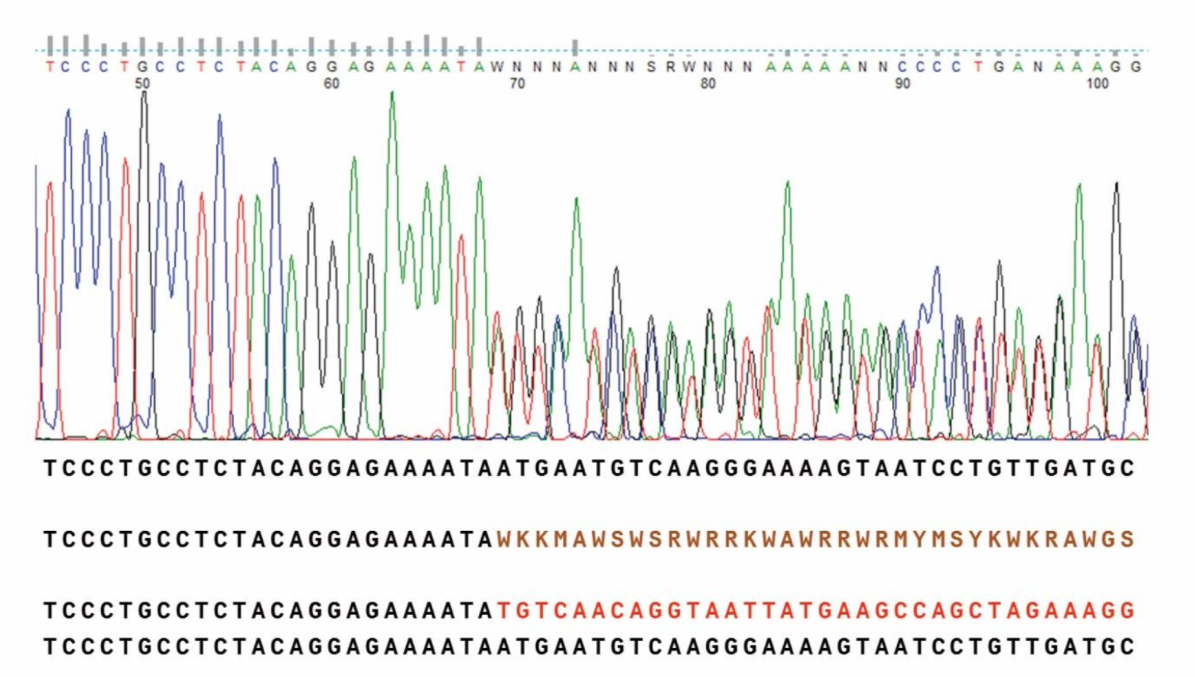

Figure S5 - Example of sequence analysis. Clone 3D7 is a clear example of a properly heterozygous clone for a larger insertion. In the first line under the chromatogram, the wild type of sequence is shown; below, partially in brown, the sequence read. It is not possible to determine with certainty which of the duplicated reads belongs to which allele, but distribution as offered on the bottom is likely, with a correct wild-type allele, and an allele with an exact 71 bp deletion that continues with the proper genomic sequence (in red).

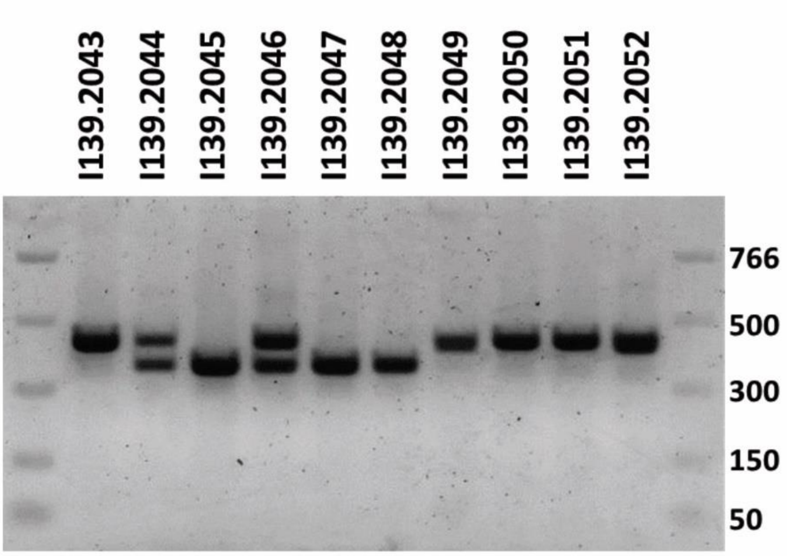

Figure S6 - Example of a PCR screening. Mice I139.2043-52 were genotyped by PCR using primers I139.5 and I139.6, using the material from each punch biopsy. Later on, homozygous animals were rebiopsied using a tail biopsy and retested. Here, I139.2043 is a wild type, 2044 a heterozygote, and 2045 a homozygote, etc. These mice are D71 derivatives; the D75 electrophoresis looks indistinguishable. The PCR material was used for sequencing in some of the samples.

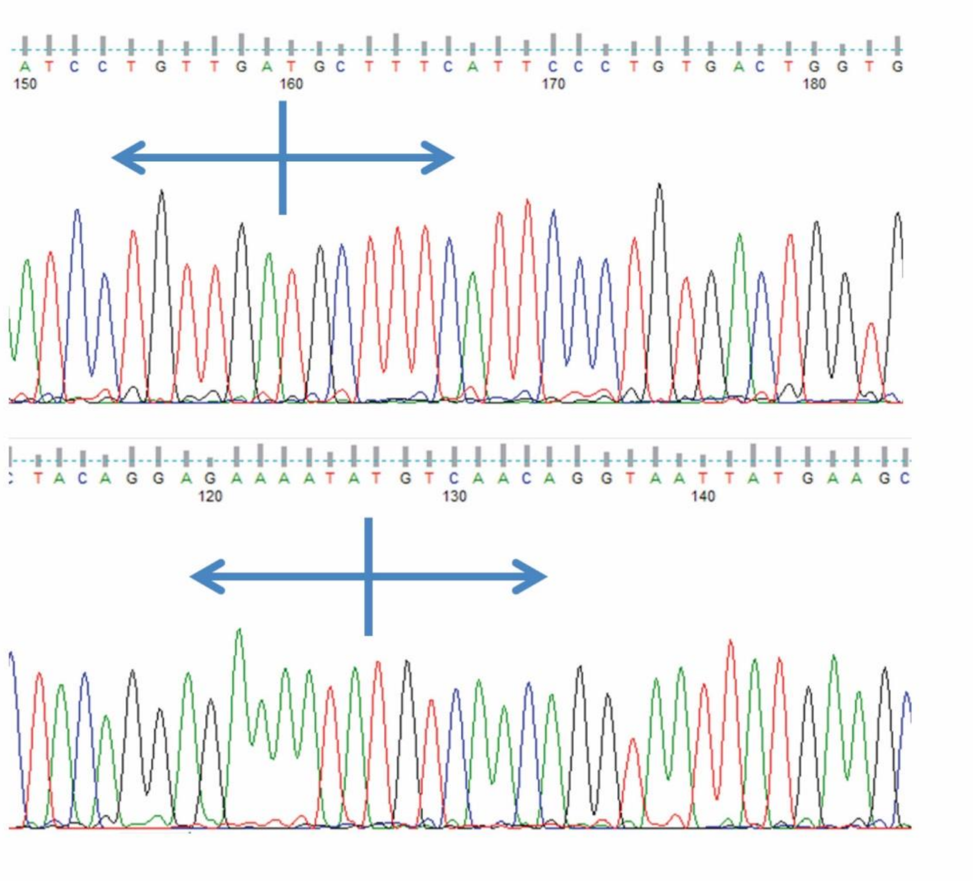

Figure S7 - Sequencing the D71 and D75 deletions in mice. Mice I139.2083 (D75 derived, top) and I139.2064 (D71 derived, bottom) were sequenced by Sanger sequencing the PCR product of I139.5/6 with sequencing primer I139.5. The two sequences confirm the ES cell data. The blue crosses delineate where the upstream sequence borders the downstream sequence (also cf. Fig. 8).

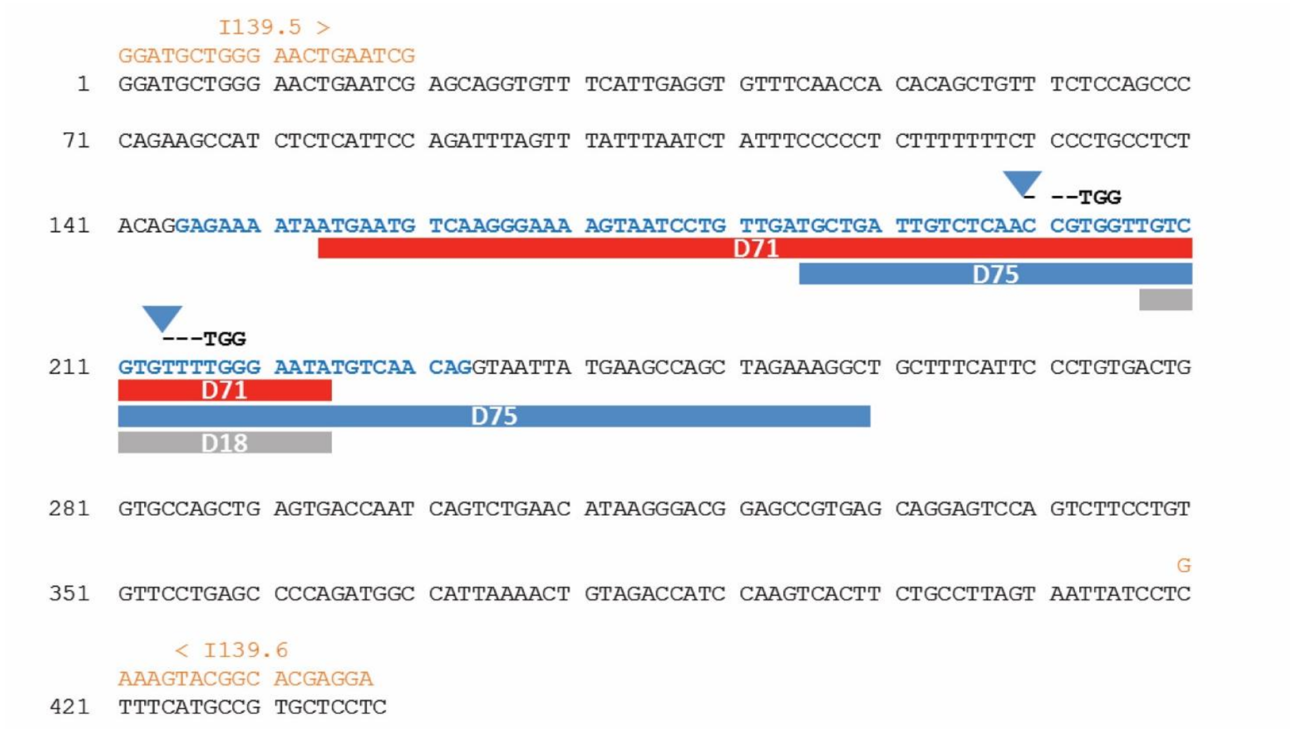

Figure S8 - Obtained mutants for Ggta1. We show the region of exon 4, with the amplification primers at the ends; the sequence in blue designates the coding sequence of exon 4. Blue triangles indicate the cut sites of the used CRISPR guides, the red (D71), blue (D75), and grey (D18) block the mapped deletions. D18 is a non-out-of-frame deletion and was not further used, and the mice were eliminated.
